# Naturally Banded Sleeve Gastrectomy Vs Non-Banded Sleeve Gastrectomy, Two Years Follow up, Controlled Clinical Trial

**DOI:** 10.1007/s11695-026-08567-8

**Published:** 2026-04-06

**Authors:** Alaa M Sewefy, Tamer E Esmaeel, Ahmed M Kamal

**Affiliations:** 1 Department of surgery, Minia University, Minya, Egypt; 2 Department of radiology, Minia University, Minya, Egypt

**Keywords:** Ring-augmented laproscopic sleeve gastrectomy, Banded laparoscopic sleeve gastrectomy, Naturally banded sleeve gastrectomy, Sewefy wrap

## Abstract

**Introduction:**

Laparoscopic sleeve gastrectomy (LSG) is the most common bariatric procedure. Although ring-augmented LSG reportedly improves long-term weight-loss outcomes, some are concerned about potential complications related to the foreign body. Therefore, this study aimed to examine 2 years of follow-up of banded sleeve with the patient’s own tissue, naturally banded LSG.

**Methods:**

This prospective randomized controlled clinical trial enrolled 80 patients. It was conducted at a university hospital between November 2022 and January 2025. Patients were randomly assigned to two equal groups: Group 1, LSG plus banding using the Teres ligament or a tight omental flap, and Group 2, LSG without banding. Patients were followed up for at least 2 years.

**Results:**

The mean follow-up period was 25.5 months. At 2 years, the mean gastric volume was significantly smaller in Group 1 (142.3 mL) than in Group 2 (218.7 mL, *p*= 0.000). Additionally, the mean percentage of excess weight loss (%EWL) was significantly higher in Group 1 (87.0%) than in Group 2 (81.8%, *p*= 0.000). Moreover, the mean percentage of total weight loss (%TWL) was significantly higher in Group 1 (42.0%) than in Group 2 (38.0%, *p*= 0.034). Finally, the mean food tolerance score was significantly lower in Group 1 (21.5) than in Group 2 (22.9, *p*= 0.000).

**Conclusions:**

Based on 2 years of follow-up, LSG with natural banding was associated with minimal gastric pouch dilatation and greater weight loss than non-banded LSG. However, long-term follow-up data are needed.

**Graphical Abstract:**

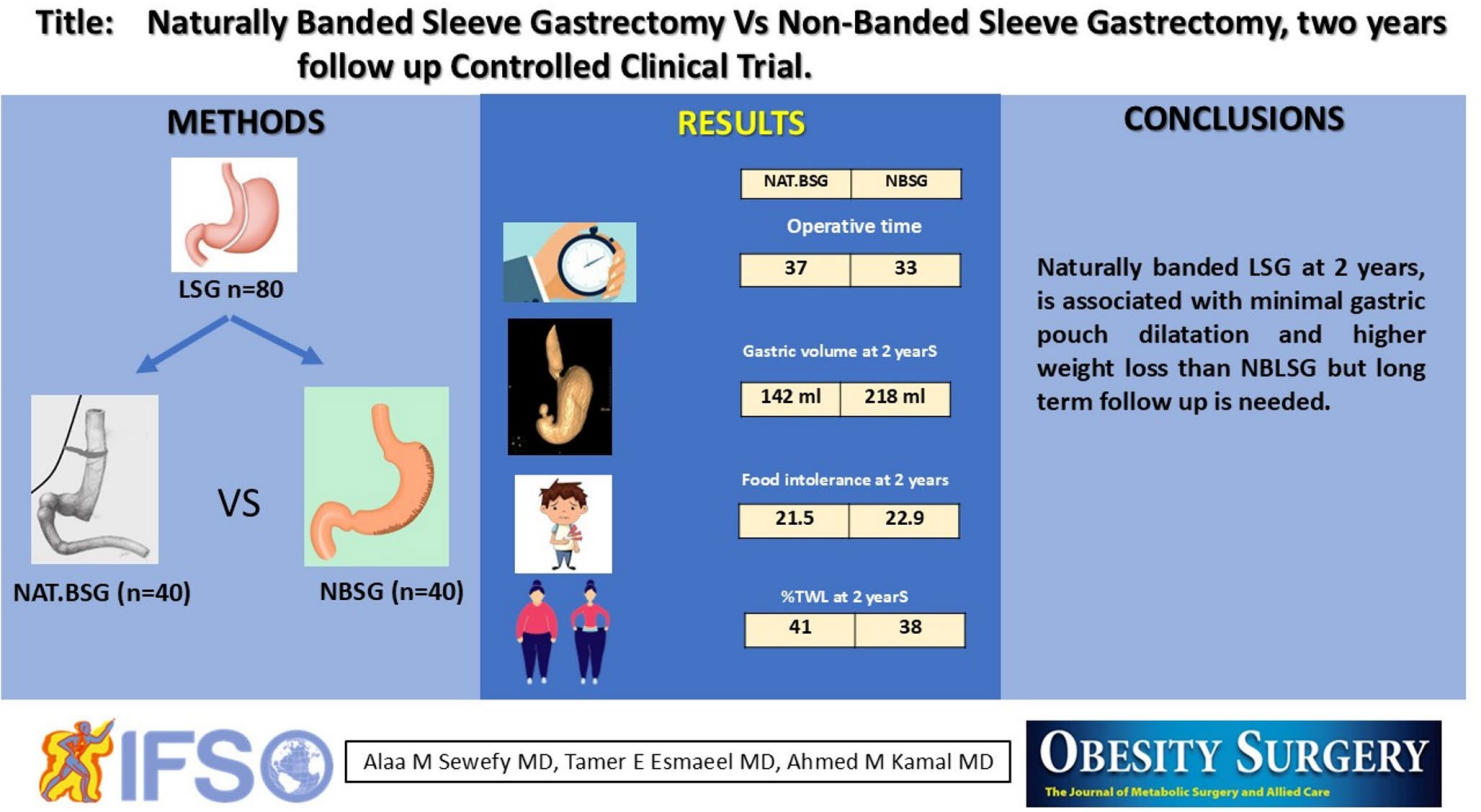

**Supplementary Information:**

The online version contains supplementary material available at 10.1007/s11695-026-08567-8.

## Key Points

LSG is associated with a high rate of long-term weight regain.

Synthetic ring augmentation reportedly improves long-term weight loss outcomes.

But it has many serious complications. We replaced it by the natural banding.

The 2 years follow up was encouraging but long follow up is needed.

**Supplementary Information:**

The online version contains supplementary material available at 10.1007/s11695-026-08567-8.

## Introduction

Obesity is a global epidemic, with about 1.9 billion adults overweight and 650 million obese. Severe obesity is associated with a higher incidence of associated diseases and short life expectancy. Bariatric surgery has proven to be the most effective long-term treatment for severe obesity [1–3].

Laparoscopic sleeve gastrectomy (LSG) is the most common bariatric procedure. Although this procedure is effective in the short term, it is reportedly associated with long-term complications, including a high incidence of gastroesophageal reflux disease (GERD) and weight regain (WR). Systematic reviews on these two issues have reported WR rates ranging from 5.7% at 2 years to 75.6% at 6 years, with a pooled incidence of new-onset GERD symptoms of 20.0%. The proposed causes of WR include initial sleeve size, sleeve dilation, increased ghrelin levels, inadequate follow-up, and personal behaviors. Blunting of the His angle, decreased gastric compliance, and increased intragastric pressure all contribute to GERD and, in some cases, even to Barrett’s esophagus (BE) after LSG [1–5].

Another technical issue that increases the risk of GERD after LSG is a radical antrectomy, which can improve weight loss but is associated with a higher incidence of GERD [6]. For all these reasons, literature recommend a close follow up for any patients with GERD symptoms and to do upper gastrointestinal endoscopy for early diagnosis and treatment [4, 5].

Rapid weight loss after LSG is also known to be a risk factor for gallstones, with incidence ranging from 0.9% to 7.5%. The best strategy to prevent gallstone formation remains controversial. Different strategies have been used, including postoperative treatment with ursodeoxycholic acid (UDCA) or concomitant cholecystectomy in patients at high risk of gallstones [7].

An increase in stomach volume over time is identified as one cause of WR after LSG, especially in patients with severe obesity, who usually require redo surgery after 3–5 years for WR [1–3, 8]. To address the issue, many studies have discussed the efficacy of adding a synthetic band to LSG to minimize WR, finding that banded LSG (BLSG) is associated with smaller pouch dilatation and WR over time compared to non-banded LSG (NBLSG). However, literature reviews have shown an average complication rate of 4%–5%, primarily patient incompatibility (2%), followed by slipping (1%), stenosis (1%), and erosions (< 0.5%) [9–12].

In our previous study, to avoid synesthetic ring complications, we used a natural flap (Teres ligament or an omental flap) to band the sleeved stomach (natural BLSG), reporting encouraging 1-year follow-up results [13, 14]. The Teres ligament and omental flap are used in various surgical procedures. The omental flap is commonly used in reconstructive surgery. The Teres ligament flap is used to repair hiatus hernias, especially with LSG. It was also reportedly used as a band in bariatric surgery early in 1996, but only for a limited number of cases and with very short follow-up (only 2 months), with no data reported after that date [15–17]. Therefore, this study aimed to evaluate 2 years of follow-up after LSG using the Teres ligament or omental flap as a natural band wrapped around the sleeved stomach to prevent gastric pouch dilatation.

## Materials and Methods

### Study Design

This prospective randomized controlled trial (registration ID: NCT05603338) was conducted at a university hospital between November 2022 and February 2025. It included all patients eligible for LSG who completed at least 2 years of follow-up. The study protocol was approved by the Faculty of Medicine, Minia University Ethical Committee (approval number 1062/11/2022). In the original approved protocol, only the use of the omentum as a natural band was planned. After the study began, we shifted to using the Teres ligament flap due to its strong ligamental component; however, in some patients with a very short Teres ligament, we had to use a tight omental flap. The ethics committee approved this amendment. All study procedures were conducted in accordance with the ethical standards of the 1964 Helsinki Declaration and its later amendments, as well as with those of the research committee [18].

### Participants

**Fig. 1:**
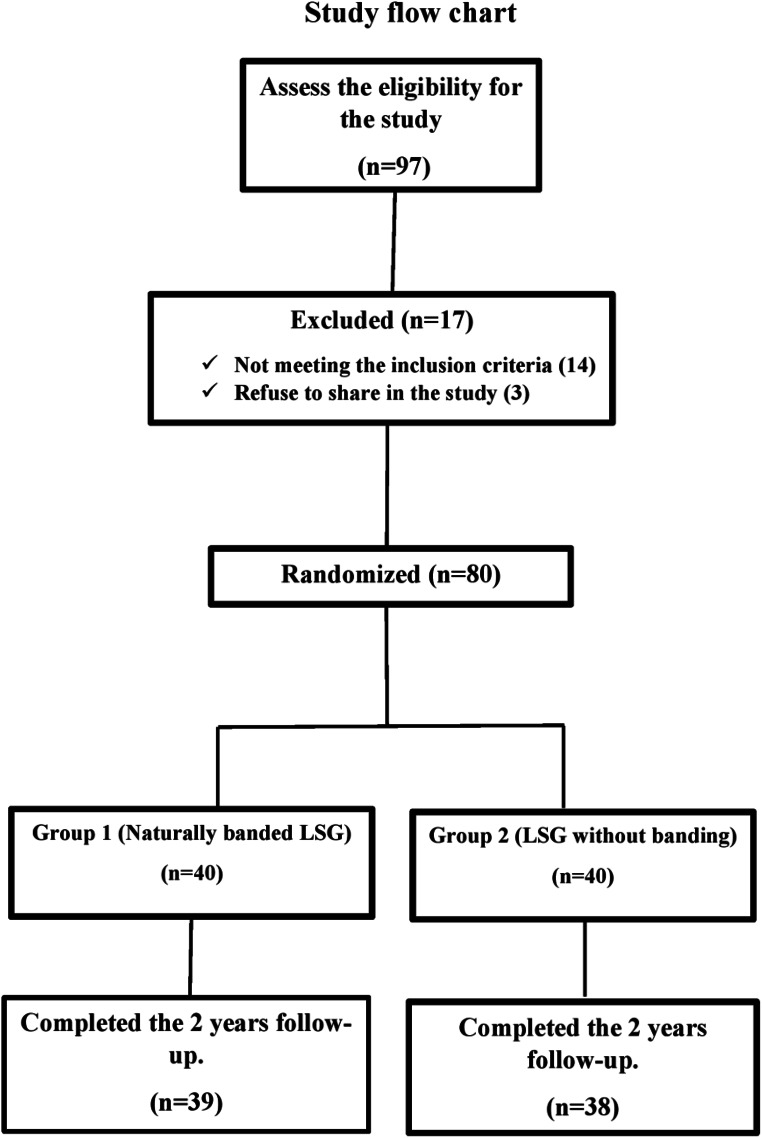
The study flowchart

The inclusion criteria were age 18–65 years and a body mass index (BMI) ˃35 kg/m^2^ without associated medical problems or > 30 kg/m^2^ with associated medical problems. The exclusion criteria were prior weight-loss surgery, preoperative GERD symptoms, inflammatory bowel disease, pregnancy, or refusal to participate in this study. The patients invited to participate in this study were provided with details about each surgical procedure, including the risks and benefits. All patients included in this study provided written informed consent. The study flowchart is shown in Fig. 1: The study flowchart.

### Randomization and Blinding

Patients were randomized using permuted block randomization; the allocation sequence was generated and concealed in sealed opaque envelopes, and the block size was variable. The patients, data analysts, radiologists, and outcome assessors were blinded to the group allocations.

### Intervention

The patients were divided into two groups of 40 each. In Group 1, patients underwent LSG, and their stomachs were then banded using a natural flap (Teres ligament or tight omental flap). In Group 2, patients underwent standard LSG without banding.

### Study Outcomes and Endpoints

The primary outcome was weight loss at two years, evaluated as the percentage of total weight loss (%TWL) at the 2-year follow-up, calculated as [(preoperative body weight − follow-up body weight)/preoperative body weight] × 100, and percentage of excess weight loss (%EWL) at the 2-year follow-up, calculated as ([initial body weight − follow-up body weight]/[initial body weight – body weight at a BMI of 25 kg/m^2^]) × 100 [19].

The secondary outcomes were sleeve size at the 2-year follow-up, evaluated by gastric volumetry using multi-detector computed tomography (MDCT); operative time, measured from port site incision to closure [19]; any complications according to the Clavien–Dindo classification; and food tolerance (FT) to all types of food, evaluated using an Arabic version of a food tolerance questionnaire, with scores ranging from 1 to 27 [20].

The study endpoints were weight loss; gastric volume; complications, especially new onset GERD; and the mean FT score at the 2-year follow-up.

### Preoperative Care

Patients underwent careful evaluation of their medical history, along with examinations and basic investigations, including a complete blood count, renal and liver function tests, random blood sugar tests, glycated hemoglobin tests, electrocardiography, and respirometry as needed. Patients with moderate and sever symptoms of GERD underwent endoscopic examination. Patients were administered low-molecular-weight heparin (LMWH) 12 h before the operation.

### Surgical Procedure

The same expert consultant bariatric surgeon operated on all patients. As standard for LSG, the greater omentum was dissected from the stomach down to near the pyloric ring, then upward to clear the left crus of the diaphragm from any adhesion. Any posterior adhesions between the stomach and pancreas were completely dissected. Using a linear stapler, the stomach was divided 2–4 cm from the pylorus and continued upward using suitable reloads. Then, the staple line was invaginated using a continuous 3/0 Prolene suture. The pouch was routinely attached to the diaphragm to prevent its later migration to the chest, its middle third to the fascia attached to the pancreas, and its lower part to the nearby omentum to prevent twisting [13].

For naturally banded LSG in Group 1, the Teres ligament was first evaluated. If it was long enough and could be passed around the stomach, it was dissected from the umbilicus downward, keeping it attached to the liver. An opening was made in the lesser omentum to pass the ligament around the sleeve 4–5 cm below the esophagogastric junction, and it was sutured to itself and to the pouch containing a 36-French bougie (Video 1). If the Teres ligament was too short, an omental flap was passed around the stomach and tightly wrapped in the presence of the 36-French bougie (Video 1).

### Gastric Voltammetry

Gastric pouch volume was evaluated by MDCT volumetric analysis at 1, 12, and 24 months postoperative on 64-slice MDCT scanners. Patients fasted for 6 h before the scan. They were then given a slow intravenous bolus injection of butylscopolamine (40 mg), then asked to swallow 2–4 packs of sodium bicarbonate granules.

### Follow-up Care

Ambulation was allowed as early as possible. Patients were allowed oral fluids from 5 h postoperatively. They were given LMWH for 14 days, proton pump inhibitors (PPIs) for at least 3 months, and UDCA (500 mg/day) for 6 months. All patients were followed up weekly for the first month, then monthly either in person (by visiting the clinic) or remotely (by telephone or WhatsApp), including a Google Form. Online and remote follow-up were found to be effective and safe. The patients could visit the clinic if they had any complaints. The patients were only allowed oral fluids for the first two weeks; a soft diet was added in the third week, followed by a high-protein, low-calorie diet [13, 19, 21, 22]. Patients underwent routine follow-up investigations every 3 months and gastric volumetry at 1, 12, and 24 months postoperatively. Patients with moderate and severe symptoms of GERD underwent endoscopic examinations, as LSG is associated with a high incidence of neo-GERD and a high prevalence of Barret esophagus of about 15% at 5–10 years postoperative compared to < 2% in the general population [23, 24].

To assess GERD symptoms, all patients were asked to complete the Arabic version of the GERD Health-related Quality of Life (GERD-HRQL) questionnaire, comprising 10 questions Total GERD-HRQL scores range from 0 to 50 and were interpreted as follows: 0, no symptoms; 1–15, mild symptoms; 16–30, moderate symptoms; and 31–50, severe symptoms. GERD was diagnosed based on GERD-HRQL scores, patients’ response to acid suppression, and upper endoscopy in patients with moderate and severe symptoms [5, 23, 25].

### Sample Size Estimation

The required sample size was estimated using G*Power (version 3.1.9.7). Studies comparing BLSG to NBLSG have reported a mean %EWL at 1 year ranging from 52% to 77% (mean: 64.2% ± 15.6%) for BLSG and from 41% to 61% (mean: 54.0% ± 14.9%) for NBLSG. Considering a two-tailed independent samples *t*-test with equal allocation, significance level of 0.05, and power of 80%, the effect size was estimated as 0.668. Based on these data, the minimal sample size was estimated as 74 patients (37 per group). Considering a dropout rate of up to 10%, 40 patients were included in each group [10, 26–29].

### Data Collection

The collected data included preoperative demographic and clinical characteristics (age, sex, height, weight, associated medical problems, and presence of gallstones), operative time, complications, improvement in the associated medical problems, gastric pouch size, weight loss, and FT at 2 years.

### Statistical Analysis

All statistical analyses were conducted using SPSS Statistics (version 25). The normality of continuous variables was assessed using the Shapiro–Wilk test. As they were normally distributed, they are presented as means ± standard deviations and compared between groups using Student’s *t*-test. Categorical and ordinal variables are presented as numbers (percentages) and compared between groups using the chi-square test. An alpha level was set to 5% with a significant level of 95%. Statistical significance was tested at p-value < 0.05.

## Results

### Baseline Data

This study included 80 patients, of whom 55 (68.8%) were female and 25 (31.2%) were male. Among patients, the mean age was 36 years, and the mean BMI was 48 kg/m^2^. Regarding associated medical problems, 45 patients (56.0%) had dyslipidemia, 21 (26.3%) had hypertension, 15 (18.8%) had obstructive sleep apnoea syndrome (OSAS), 9 (11.3%) had type 2 diabetes mellitus (T2DM), and 7 (8.8%) had gallstones. Preoperative characteristics did not differ significantly between the BLSG and NBLSG groups. The mean follow-up time was 25.5 months (Table 1).

**Table 1:** Preoperative and operative and follow up data

		Total (80)	Group		Group 2 (Nonbanded) (*n* = 40)	*P* value
			Group 1(Banded) (*n* = 40)			
			Teres ligament (*n* =33_	Omentum(*n* = 7)		
Sex	**Female**	55 (68.8%)	27 (67.5%)		28 (70%)	0.809
	**Male**	25(31.2%)	13 (32.5%)		12 (30%)	
Age in year		36.3 ± 12.4	36.6 ± 12.50		36 ± 12.5	0.420
BMI (kg/m2)		47.9 ± 5.7	47.4 ± 5.8		47.1 ± 7.6	0.723
Gallstone		7 (8.8%)	3 (7.5%)		4 (10%)	0.692
DM ^*^		9 (11.3%)	5 (12.5%)		4 (10%)	0.723
HPN ^≠^		21 (26.3%)	10 (25%)		11 (27.5%)	0.799
Hyperlipidemia		45(56.3%)	21(52.5%)		24(60%)	0.499
OSAS ^$^		15 (18.8%)	8 (20%)		7 (17.5%)	0.775
Operative Time in minutes			37 ± 4		33 ± 5	< 0.001

Quantitative data were presented by mean and standard deviation, while qualitative data were presented by frequency distribution. Student’s t-test was used to compare continuous variables, and the Chi-square test was used to compare categorical or ordinal variables. An alpha level was set to 5% with a significant level of 95%. Statistical significance was tested at p-value < 0.05

*Diabetes Meletus, ≠Hypertension, $ Obstructive sleep apnea syndrome

### Operative Data

All patients who had preoperative gallstones also underwent cholecystectomy during the same surgery. The mean operative time was significantly longer in Group 1 (37 ± 4 min) than in Group 2 (33 ± 5 min, *p* = 0.000). In Group 1, the Teres ligament was used as a band in 33 (82.5%) patients, whereas a tight omental flap was used in 7 (17.5%, Table 1).

### Weight Loss

**Fig. 2:**
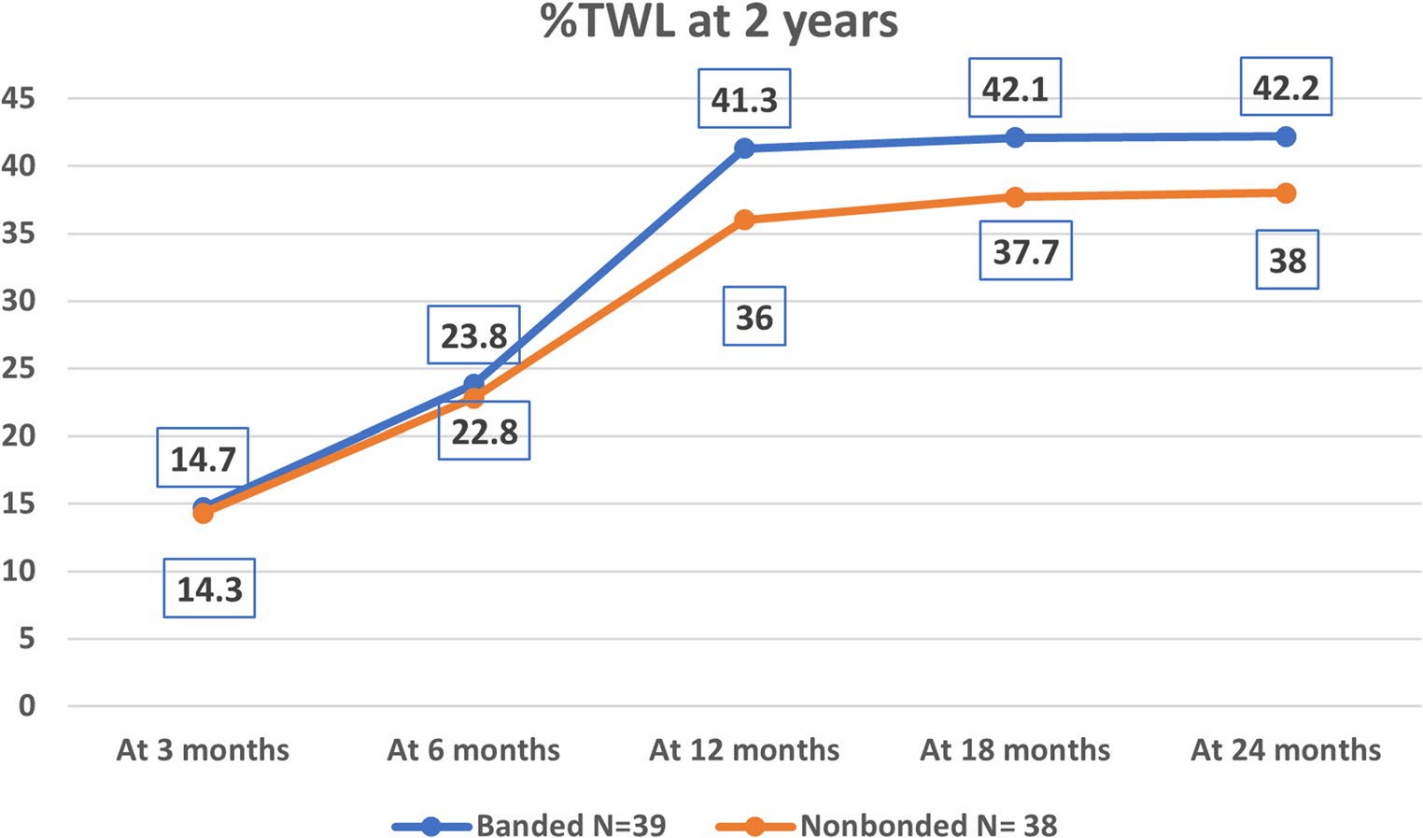
Line chart show %TWL over 2 years

**Fig. 3:**
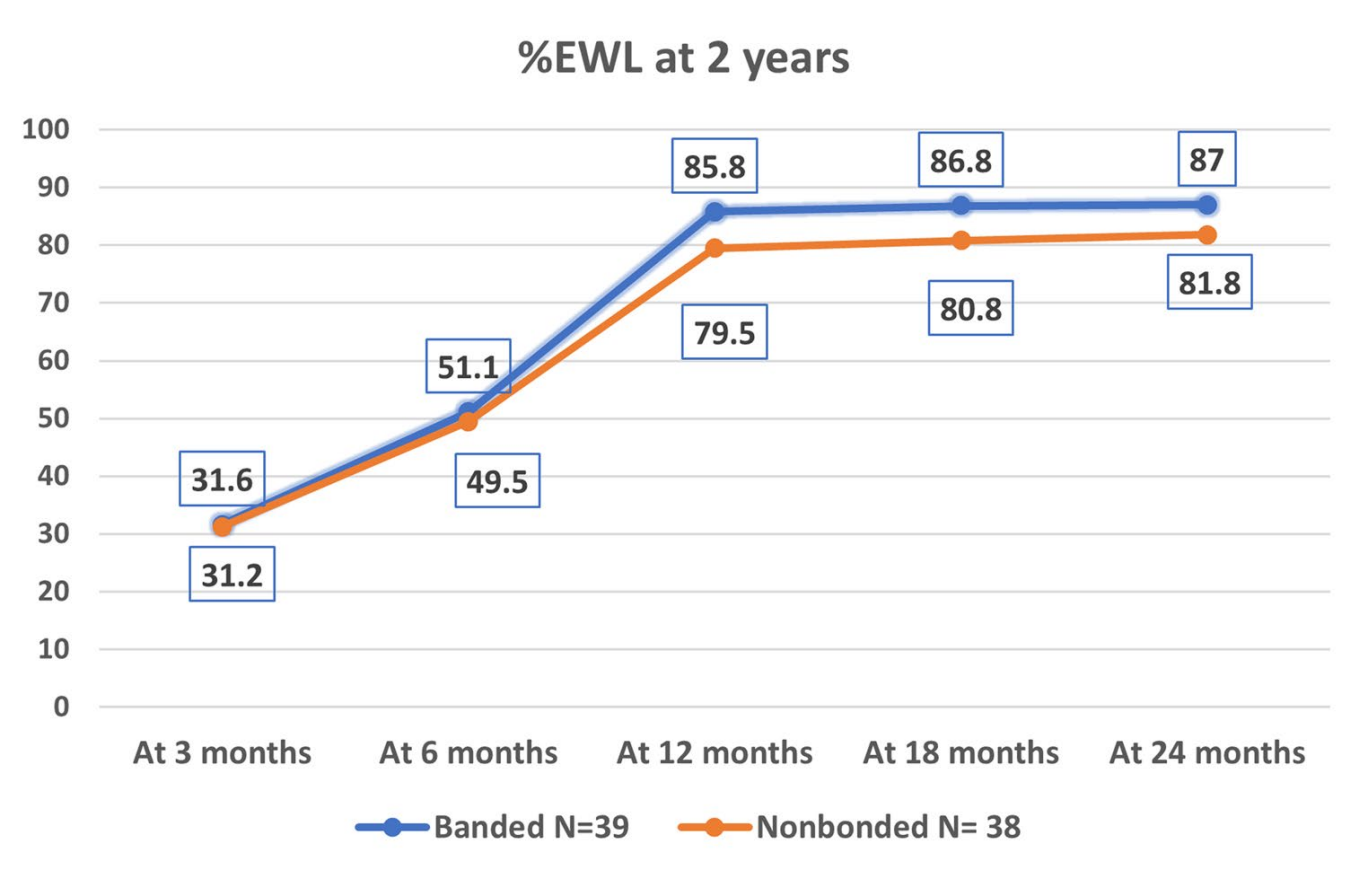
Line chart show %EWL over 2 years

Weight loss is presented by group and time point in Table 2. Weight loss at 3 and 6 months postoperative did not differ significantly between groups. In contrast, the %TWL at 2 years was significantly greater in Group 1 (42.0% ± 6.6%) than in Group 2 (38.0% ± 5.6%, *p* = 0.034), see Fig. 2: %TWL over 2 years. Similarly, the %EWL at 2 years was significantly greater in Group 1 (87.0% ± 5.3%) than in Group 2 (81.8% ± 5.8%, *p* = 0.000), see Fig. 3: %EWL over 2 years.

**Table 2:** Weight loss parameters over 24 months

		Group 1 (Banded) *N* = 39	Group 2 (Nonbonded) *N* = 38	P value
%TWL3	At 3 months	14.7 ± 2.3	14.3 ± 2.4	0.402
	At 6 months	23.8 ± 3.1	22.8 ± 3.1	0.140
	At 12 months	41.3.6.3	36 ± 5.6	0.010
	At 18 months	42.1 ± 7.5	37.7 ± 7.6	0.012
	At 24 months	42.2 ± 6.6	38 ± 5.6	0.034
**%EWL3**	At 3 months	31.6 ± 5	31.2 ± 6	0.728
	At 6 months	51.1 ± 6.8	49.5 ± 6.2	0.282
	At 12 months	85.8 ± 8.7	79.5 ± 8.4	0.002
	At 18 months	86.8 ± 5.1	80.8 ± 5.6	0.000
	At 24 months	87 ± 5.3	81.8 ± 5.8	0.000

Data were presented by mean and standard deviation. Student’s t-test was used to compare variables. Statistical significance was tested at p-value < 0.05

%*EWL* percentage of excess weight loss, *BMI* Body mass index, %*TWL* percentage of total weight loss

### Gastric Volume

Gastric volumes are presented by group and time point in Table 3. The mean gastric volume at 1 month did not differ significantly between Group 1 (96 ± 3 mL) and Group 2 (95 ± 5 mL). In contrast, mean gastric volume at 2 years was significantly greater in Group 2 (218.7 ± 38.3 mL) than in Group 1 (142.3 ± 12.4 mL, *p* = 0.000).

**Table 3:** Gastric volume over 24 months

	Group 1(Banded) *N* = 39	Group 2 (Nonbonded) *N* = 38	*P* value
Gastric volume at 1 month	96 ± 3	95.2 ± 5.	0.470
Gastric volume at 12 months	112.8 ± 4.5	147.3 ± 10.3	0.000
Gastric volume at 24 months	142.3 ± 12.4	218.7 ± 38.4	0.000

Data were presented by mean and standard deviation. Student’s t-test was used to compare variables. Statistical significance was tested at p-value < 0.05

### Complications

Complications are presented by group in Table 4. Intramural bleeding occurred in one (2.6%) patient in Group 1, as evidenced by melena and hematemesis on the second day postoperative, who was managed conservatively. One (2.6%) patient developed deep vein thrombosis (DVT) in Group 2 and was managed with a therapeutic dose of LMWH. One (2.6%) patient in Group 1 and two (5.3%) patients in Group 2 developed anemia (*p* = 0.541). Three (7.7%) patients in Group 1 and one (2.6%) patient in Group 2 developed vitamin D deficiency (*p* = 0.317). Five (12.8%) patients in Group 1 and three (7.9%) patients in Group 2 developed neo-GERD (*p* = 0.479). One (2.6%) patient in Group 1 was converted to Roux-en-Y gastric bypass (RYGB) due to intractable GERD.

**Table 4:** Complications

Type of Complications		Group		Grade	*P* value
		Group 1 (Banded) *N* = 39	Group 2 (Nonbonded) *N* = 38		
Bleeding		1 (2.6%)	0%	Grade II	0.320
DVT		0%	1 (2.6%)	Grade II	0.320
Nutritional	Anemia	1(2.6%)	2 (5.3%)	Grade II	0.541
	Vitamin D def.	3 (7.7%)	1 (2.6%)	Grade II	0.317
De novo GERD		5 (12.8%)	3 (7.9%)	Grade II- Grade III	0.479

Data were presented by frequency distribution. Chi-square test was used to compare variables. Statistical significance was tested at p-value < 0.05.

Complication according to the Clavien–Dindo classification.

DVT: Deep venous thrombosis, GERD: gastro-esophageal reflux disease

### FT

**Fig. 4:**
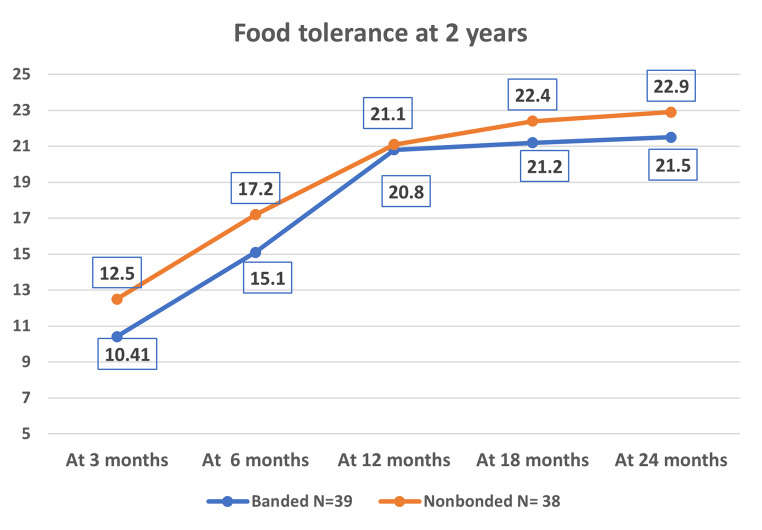
Line chart show food tolerance over 2 years

FT scores are presented by group and time point in Table 5. The FT scores at 3 and 6 months were significantly lower in Group 1 than in Group 2. However, FT scores at one year did not differ significantly between Group 1 (20.8 ± 0.9) and Group 2 (21.1 ± 0.1, *p* = 0.172). Nonetheless, FT scores at 2 years were again significantly lower in Group 1 (21.5) than in Group 2 (22.9, *p* = 0.000) due to greater dilatation of the gastric pouch in Group 2 compared to Group 1, see Fig. 4: The food tolerance score over 2 years.

**Table 5:** Food tolerance over 24 months

Food tolerance	Group 1 (Banded)	Group 2 (Nonbonded) *N* = 38	*P* value
At 3 months	10.41 ± 1.5	12.5 ± 2	0.000
At 6 months	15.1 ± 1.1	17.2 ± 1.1	0.000
At 12 months	20.8 ± 0.9	21.1 ± 1.1	0.172
At 18 months	21.2±0.8	22.4±0.6	0.000
At 24 months	21.5 ± 0.6	22.9 ± 0.3	0.000

Data were presented by mean and standard deviation. Student’s t-test was used to compare variables. Statistical significance was tested at p-value < 0.05

### Remission of associated medical problems

Changes in associated medical problems are presented by group in Table 6. T2DM went into remission in 80.0% patients in Group 1 compared with 75.0% patients in Group 2 (*p* = 0.858). OSAS went into remission in 75.0% patients in Group 1 compared with 71.4% in Group 2 (*p* = 0.876). Hypertension went into remission in 70.0% patients in Group 1 compared with 72.7% in Group 2 (*p* = 0.890). Hyperlipidemia went into remission in 61.9% patients in Group 1 compared with 70.8% in Group 2 (*p* = 0.526).

**Table 6:** Improvement of associated medical problems

	Banded			Non banded			*P* value
	Pre.	Post.	% of Remission	Pre.	Post.	% of Remission	
DM*	5	1	80%	4	1	75%	0.858
HPN ^≠^	10	3	70%	11	3	72.7%	0.890
OSAS ^$^	8	2	75%	7	2	71.4%	0.876
HLP ^¥^	21	8	61.9%	24	7	70.8%	0.526

qualitative data were presented by frequency distribution. The Chi-square test was used to compare categorical or ordinal variables. An alpha level was set to 5% with a significant level of 95%. Statistical significance was tested at p-value < 0.05

*Diabetes Meletus, ≠Hypertension, $ Obstructive sleep apnea syndrome, ¥ Hyperlipidemia

## Discussion

Although LSG is the most common bariatric operation, its primary drawback is that the pouch dilatates over time, leading to WR. Gastric volumetry is reported to be the best modality for assessing sleeve volume [30, 31]. In our study, the mean gastric volume was 96.0 ± 3.2 mL with naturally banded LSG and 95.0 ± 5.7 mL with NBLSG at 1 month postoperative, increasing at 2 years following both procedures (142.3 ± 12.4 and 218.7 ± 38.3 mL, respectively), indicating significant pouch dilatation with NBLSG. Ali et al. reported that following NBLSG, gastric volume ranged from 60 to 107 mL at 1 month (median: 82.9 mL), with a mean gastric volume of 171.6 mL at 1 year [30]. Hany et al. reported a mean gastric volume at 1 year of 177.6 mL following NBLSG and 111 mL following BLSG [11]. Braghetto et al. reported gastric volumes of 116 mL in the early postoperative period and 254 mL at 2 years following NBLSG [31].

In our study, the %TWL and %EWL at 2 years were significantly higher after naturally banded LSG (42.0% and 87.0%, respectively) than after NBLSG (38.0% and 81.8%, respectively). Most studies comparing BLSG to NBLSG have reported significantly greater weight loss following BLSG than NBLSG [11, 26, 32–37]. They have reported %EWL at 1 year of 52%–77% following BLSG compared with 41%–61% following NBLSG. In our study, weight loss was greater due to a small bougie size, sleeve plication, and greater antral resection, which have been reported to improve weight loss [6, 38–40].

In our study, the mean operative time was 33 ± 5 min for NBLSG compared to 37 ± 4 min for naturally banded LSG. Previous studies have reported a mean operative time of 49–87 min for BLSG compared with 46–75 min for NBLSG. The shorter operative times in our study can be explained by all patients being operated on by the same expert senior surgeon with extensive experience in the field. Operative time is known to decrease with a surgeon’s experience; for example, there is a reported reduction of 40 min in the operative time by Carandina et al. in early and late experience of LSG [41–43].

Food intolerance is the most commonly reported consequence of BLSG. In our study, the mean FT score at 2 years was significantly lower following naturally banded LSG (21.5) than NBLSG (22.9). Hany et al. reported a mean FT score of 21.46 among patients who underwent BLSG and 21.43 among patients who underwent NBLSG at 1 year; however, after 4 years, the mean FT score was significantly higher among those who underwent BLSG (24.00) but remained unchanged among those who underwent NBLSG (21.00) [11]. However, many studies have found that food intolerance is more common following BLSG than NBLSG [1, 28, 33, 35].

In our study, the incidence of neo-GERD was 12.8% following naturally banded LSG compared with 7.9% following NBLSG. In contrast, a systematic review and meta-analysis by Chaouch et al. reported an incidence of neo-GERD to be 13.8% following BLSG and 19.6% following NBLSG [1]. Similarly, Hany et al. reported an incidence of neo-GERD of 20.2% following NBLSG compared with 18.9% following BLSG [11].

At the beginning of this study, the main criticism from the bariatric surgeon was the durability of the natural band, which was expected to loosen and lose function as patients lose weight. However, for two patients who had gallstones more than 18 months postoperatively and underwent laparoscopic cholecystectomy, we checked the band, confirming that it had not loosened but was adhered to the stomach without any apparent gastric dilatation (Video 2).

### Study Limitations

Our study had several limitations that should be acknowledged. Firstly, it only compared BLSG with a natural band to NBLSG. Its conclusions would have been strengthened by including a third group in which BLSG is performed using a synthetic band, enabling a comparison of weight loss and complications between BLSG with the two banding methods. Secondly, in Group 1, the omentum was used as a band in 7 patients and the Teres ligament in 33 patients. However, it would have been better if Group 1 had been divided into two equal subgroups, which would have enabled a comparison of the two types of natural banding. Thirdly, because one surgeon operated on all patients, our results may not generalize to other surgeons who perform these techniques. Indeed, not all surgeons make the same sleeves, such as 32 versus > 40 French nasogastric tubes and invagination staple line versus reinforcement. Finally, the follow-up time was limited to 2 years. Therefore, long-term data are required to further validate this procedure.

## Conclusions

Based on 2 years of follow-up data, BLSG using natural bands was associated with minimal gastric pouch dilatation and greater weight loss than NBLSG. However, long-term follow-up data are needed to further validate this approach.

## Supplementary Information

Below is the link to the electronic Supplementary Material.

Supplementary Material 1 (DOC 218 KB)

Supplementary Material 2 (MOV 85.1 MB)

Supplementary Material 3 (MP4 428 MB)

## Data Availability

The data that support the findings of this study are available on request from the corresponding author on reasonable request.
